# Medical ozone treatment for pain, fatigue, anxiety, and depression in cancer patients: a scoping review

**DOI:** 10.3389/fpsyg.2025.1687754

**Published:** 2025-11-18

**Authors:** Lei Lei, Xiaojing Xue, Gang Feng, Xiaoyan Wang, Yuan Jiang

**Affiliations:** Department of Oncology, Mianyang Central Hospital Affiliated Hospital of Electronic Science and Technology University, Mianyang, China

**Keywords:** cancer, medical ozone treatment, symptoms, scoping review, ozone

## Abstract

**Aim:**

The aim of this study is to conduct a scoping review on the application of medical ozone treatment in managing symptoms (including Pain, Fatigue, Anxiety, and Depression) among cancer patients, aiming to provide clinical medical staff with a reference for implementing a novel approach to symptom management.

**Methods:**

This scoping review was conducted based on the methodology presented by Arksey and O’Malley and The Joanna Briggs Institute methodology was employed to conduct scoped reviews of Pubmed, Web of science, CINAHL, EMBASE, MEDLINE, APA and CNKI in order to address the research question: “What is the impact of medical ozone treatment on pain, fatigue, anxiety, and depression in cancer patients?" Two independent reviewers select samples by screening and reviewing articles and extract data using a data chart form. The protocol was registered in the Open Science Framework (OSF.)

**Results:**

A total of 16 articles were included to analyze and integrate the use of medical ozone, medical ozone dose, symptom assessment tools, and symptom management.

**Conclusion:**

The effectiveness of medical ozone treatment in managing key symptoms (including Pain, Fatigue, Anxiety, and Depression) among cancer patients has been preliminarily verified. Future studies need to cover a wider range of areas and larger sample sizes, as well as conduct long-term follow-up studies and multi-dimensional evaluation to enhance the universality and reliability of the results.

## 1 Background

The diagnosis and treatment of cancer continue to advance, leading to improved survival rates for patients. However, with the increase in survival rates, there has also been a rise in the incidence of adverse effects experienced by cancer survivors due to cancer treatment. Among these adverse effects, anxiety, depression, pain, and fatigue are commonly reported symptoms in cancer survivors (Koo et al., 2020). The study revealed that 17 and 9% of cancer survivors reported moderate to severe levels of anxiety and depression, respectively (Götze et al., 2020). Additionally, over half of the cancer survivors experienced moderate to severe pain throughout the 5-year survival period (Bennett et al., 2017). These adverse symptoms contribute to a decline in quality of life and an increase in distress among cancer survivors.

Ozone (O_3_), an allotrope of oxygen that consists of three oxygen atoms, is an unstable, light-blue gas with high oxidizing potential. Since it was formally defined in 1840, it has a history of nearly 200 years of research, application, and development in various fields. In medicine, it is recognized as a potent oxidizing and antimicrobial agent (Bocci et al., 2009). Medical Ozone Treatment has consequently been utilized as a complementary or adjunctive therapy for a range of conditions, including infectious, cardiovascular, and gastrointestinal diseases, as well as osteoarthritis. Its clinical applications include the prevention and management of dental caries by controlling oral pathogens (Sen and Sen, 2020), and the alleviation of lumbar spine pain in adults. Research suggests that medical ozone treatment can be an effective option for relieving lumbar spine pain (Andrade et al., 2019). Furthermore, its potential role as an adjunctive therapy in anti-tumor treatment is currently under investigation. Studies have explored its potential to enhance the efficacy of radiotherapy in esophageal cancer (Guo et al., 2024) and its use as ozonated water to manage chemotherapy-induced stomatitis (Hayashi et al., 2019). Most relevant to this review, several studies highlight the potential of medical ozone treatment in managing cancer-related symptoms, such as fatigue, anxiety, depression, and pain. However, as this review synthesizes, a significant gap remains in the establishment of standardized, guideline-driven protocols for its application in managing this specific cluster of symptoms (Elvis and Ekta, 2011).

An initial search of PubMed, Cochrane, CINAHL, Embase, Scopus, Web of Science, CNKI, and MEDLINE was conducted up to October 1, 2025. This search confirmed that no prior scoping reviews on this topic exist. This gap in the literature justifies the present scoping review, which aims to map the evidence regarding the effect of medical ozone treatment on symptom management in cancer patients. The findings of this review are intended to inform future research and guide clinical practice.

## 2 Ethics and dissemination

This review does not require ethical approval as it is a secondary analysis of pre-existing, published data.

## 3 Methods

This study is a scoping review, which follows JBI method (Peters et al., 2020) and Scoping Reviews (PRISMA-ScR) guidelines (Tricco et al., 2018). The review protocol was registered in the Open Science Framework.^1^

### 3.1 Research question

To formulate the guiding question, the acronym PCC was used, in which “P” represents the population (Cancer patients); “C” the concept (Medical Ozone Treatment); and “C” the context (Broad, without restriction). Thus, the guiding question of this study was: What is the impact of medical ozone treatment on pain, fatigue, anxiety, and depression in cancer patients?

### 3.2 Inclusion and exclusion criteria

Inclusion:

- Study Types: Studies published in full, with no restrictions on methodological design, languages or time limits.
- Participants: Adult patients (age ≥ 18 years) with a diagnosis of cancer, regardless of cancer type or stage.
- Intervention: Studies investigating Medical Ozone Treatment as a primary or adjuvant intervention, regardless of administration route or dosage.

Exclusion:

- Studies not published in full text.
- Studies where the full text is unavailable despite exhaustive search efforts.

### 3.3 Search strategy

After defining the PCC acronym elements, two medical librarians made the search strategy in DeCS, MeSH and Emtree vocabularies. Searches in databases were created on October 5, 2025 with the help of a librarian, according to the sets of terms in Table 1.

**Table 1:** Search strategy.

Search strategy	
1#	Search:"Neoplasms”[MeSH Terms] OR “Neoplas*”[Title/Abstract] OR “Tumor”[Title/Abstract] OR “Cancer”[Title/Abstract] OR “Malignanc*”[Title/Abstract])OR “Oncolog*”[Title/Abstract] OR “Carcinom*” [Title/Abstract]OR “Sarcoma*” [Title/Abstract]OR “Adenocarcinom*”[Title/Abstract] OR “Metastasis” OR “Metastatic”[Title/Abstract])
2#	Search:"Depress*”[MeSH Terms] OR “Depression “[Title/Abstract] OR “Depressive disorder”[Title/Abstract] OR “Depressive symptoms”[Title/Abstract] OR “Major depressive disorder”[Title/Abstract] OR “Anxiety”[MeSH Terms] OR “Anxiety disorder”[Title/Abstract] OR “Generalized anxiety disorder”[Title/Abstract] OR “Fatigue”[MeSH Terms] OR “Fatigue sydrome “[Title/Abstract] OR “Mental fatigue”[Title/Abstract] OR “Pain”[MeSH Terms] OR “Pain management “[Title/Abstract] OR “Cancer pain “[Title/Abstract] OR “Chronic pain”[Title/Abstract] OR “Quality of Life”[Mesh] OR “Health-Related Quality of Life”[Title/Abstract] OR “QoL”[Title/Abstract] OR “Symptoms”[Mesh] OR Symptom*[Title/Abstract] OR “Symptom cluster”[Title/Abstract] OR “Symptom management”[Title/Abstract]
3#	Search:"Ozone”[Mesh] OR “Ozone Therapy”[Title/Abstract] OR “Medical Ozone”[Title/Abstract] OR “Medical Ozone Treatment”[Title/Abstract] OR “Oxygen-Ozone Therapy”[Title/Abstract] OR “Ozonotherapy”[Title/Abstract] OR “Autohemotherapy”[Title/Abstract] OR “Ozonated Autohemotherapy”[Title/Abstract] OR “Rectal Insufflation”[Title/Abstract] OR “Ozone Insufflation”[Title/Abstract]
4#	Search: #1 AND #2 AND #3

## 4 Results

### 4.1 Literature screening results

**Figure 1:**
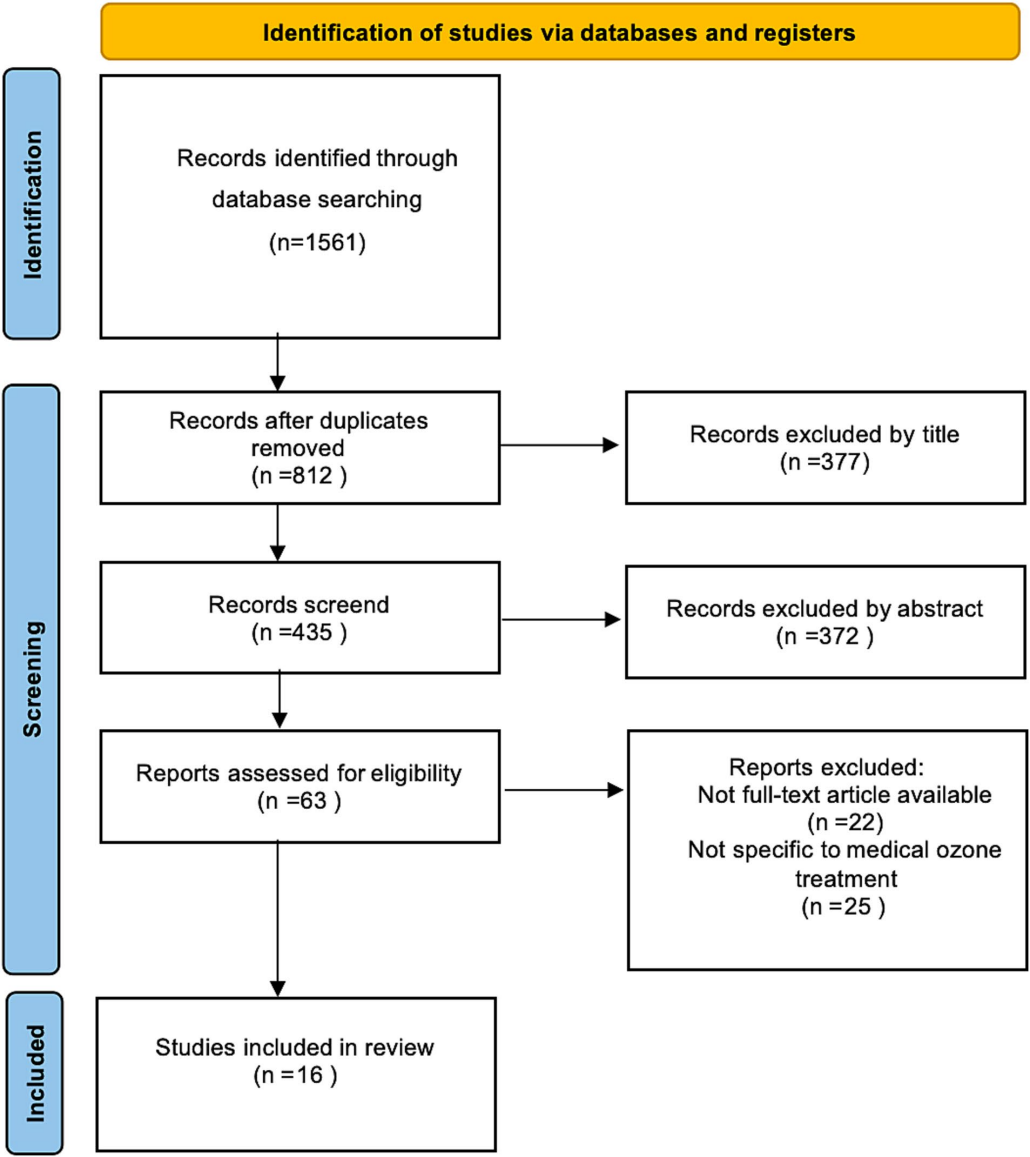
PRISMA flow chart for the scoping review process.

The search across all databases initially identified 1,561 records. Following the removal of 749 duplicates, 812 unique records were screened based on their titles and abstracts. After this initial screening, the full text of the remaining articles was assessed for eligibility according to the pre-defined criteria. This process resulted in the inclusion of 16 studies. Figure 1 presents the PRISMA flow diagram, illustrating the complete study selection process and the reasons for exclusion at each stage.

The characteristics of the 16 included studies are summarized in Table 2. Regarding chronological distribution, the majority of studies were published in 2018 (*n* = 3) (Li and Yao, 2018; Zhang et al., 2018; Tirelli et al., 2018) and 2023 (*n* = 3; Tirelli et al., 2023; Clavo et al., 2023b; Clavo et al., 2023a). Other studies were published in 2011 (Lu et al., 2011), 2017 (Kang et al., 2017), 2020 (*n* = 2; Clavo et al., 2021b; Li, 2020), 2021 (Clavo et al., 2021a), 2022 (Clavo et al., 2022), 2024 (Su et al., 2024; Li and Pu, 2024; *n* = 2), and 2025 (*n* = 2; Clavo et al., 2025b; Clavo et al., 2025a). Geographically, nearly half of the studies (n = 7, 43.8%) originated from China, while seven (43.8%) were from Spain, and two (12.5%) were from Italy. In terms of methodological design, the majority were case series (*n* = 8, 50.0%), followed by randomized clinical trials (*n* = 6, 37.5%), and reviews (*n* = 2, 12.5%). It is noteworthy that seven of the included studies were authored by or co-authored by Bernardino Clavo from Spain.

**Table 2:** General characteristics of included literature (*n* = 16).

No.	Author	Journal/field	Year/Country	Title	Method
01	Clavo et al. (2025b)	Integr Cancer Ther	2025/Spain	Long-Term effects of ozone treatment in patients with persistent numbness and tingling secondary to chemotherapy-induced peripheral neuropathy: a retrospective study.	A case series study
02	Clavo et al. (2025a)	Cancers (Basel)	2025/Spain	Ozone treatment in the management of chemotherapy-induced peripheral neuropathy: a Review of rationale and research directions.	Review
03	Su et al. (2024)	Chin J Pain Med	2024/China	High-voltage pulse radiofrequency combined with medical ozone water for treating cancer patients with acute herpes zoster neuropathic pain after chemotherapy	Randomized Clinical Trial
04	Li and Pu (2024)	Integr Cancer Ther	2024/China	Ozone therapy for breast cancer: an integrative literature review	Review
05	Tirelli et al. (2023)	Eur Rev. Med Pharmacol Sci	2023/Italy	Oxygen-ozone autohemotherapy in breast cancer patients suffering from fatigue and musculoskeletal pain upon aromatase inhibitors treatment: a case-series study	A case series study
06	Clavo et al. (2023b)	Int J Environ Res Public Health	2023/Spain	Effects of ozone treatment on health-related quality of life and toxicity induced by radiotherapy and chemotherapy in symptomatic cancer survivors	A case series study
07	Clavo et al. (2023a)	Front Psychol	2023/Spain	Effects of ozone therapy on anxiety and depression in patients with refractory symptoms of severe diseases: a pilot study	A case series study
08	Clavo et al. (2022)	Front Physiol	2022/Spain	Long-term improvement by ozone treatment in chronic pain secondary tochemotherapy-inducedperipheral neuropathy: A preliminary report	A case series study
09	Clavo et al. (2021a)	J Palliat Med	2021/Spain	Ozone therapy in refractory pelvic pain syndromes secondary to cancer treatment: a new approach warranting exploration	A case series study
10	Clavo et al. (2021b)	Pain Medicine	2021/Spain	Long-term results with adjuvant ozone therapy in the management of chronic pelvic pain secondary to cancer treatment	A case series study
11	Li (2020)	Cardiovasc Dis J Integr Tradit Chin West Med (Electron)	2020/China	Clinical observation of medical ozone combined with oxycodone sustained-release tablets in the treatment of moderate to severe cancer pain	Randomized Clinical Trial
12	Li and Yao (2018)	China J Mod Med	2018/China	Medical ozone as an adjuvant therapy to oxycodone for moderate-to-severe Cancer Pain	Randomized Clinical Trial
13	Zhang et al. (2018)	World Latest Med Inform (Electron)	2018/China	Research progress on ozone immunotherapy for cancer-related fatigue	Review
14	Tirelli et al. (2018)	Eur Rev. Med Pharmacol Sci	2018/Italy	Oxygen-ozone therapy as support and palliative therapy in 50 cancer patients with fatigue–A short report	A case series study
15	Kang et al. (2017)	Modern Journal of Integrated Traditional Chinese and Western Medicine	2017/China	The effect of medical ozone autotransfusion on basic fibroblast growth factor and alpha-fetoprotein in elderly patients with hepatocellular carcinoma after radiofrequency ablation	Randomized Clinical Trial
16	Lu et al. (2011)	Chinese General Practice Nursing	2011/China	Nursing intervention for breast carcinoma patients with cancer—related fatigue accepting immuned ozone treatment	Randomized Clinical Trial

### 4.2 Description of systematic reviews of medical ozone treatment for cancer patients

From each included study, we systematically extracted data into predefined fields, including: tumor site, specific symptoms managed, medical ozone treatment parameters (route of administration and dosage), and assessment tools. All extracted data are summarized in Table 3.

**Table 3:** Description of systematic reviews of medical ozone treatment for cancer patients (*n* = 16).

No.	Author	Tumor site	Sample	Usage of ozone	Dose of ozone	Symptom	Assessment tool	Finding
01	Clavo et al. (2025b)	Colon and rectum; Gynecological tumors; Lung; Lymphoma; Breast; Head and neck	15	Rectal insufflation	10 ~ 30 μg/mL	Numbness and tingling secondary to chemotherapy-induced peripheral neuropathy	VAS	67% of patients reported a decrease in numbness and tingling ≥50% (*p* = 0 0.002).
02	Clavo et al. (2025a)	Not specified	/	Rectal insufflation	10 ~ 30 μg/mL	Pain	VAS	Patients in medical ozone treatment showed improvement in pain.
03	Su et al. (2024)	Thoracolumbar segment	86	Hypodermic injection	10 ~ 30 μg/mL	Pain; Anxiety; Depression	VAS; GAD-7; PHQ-9	The observation group showed significantly greater improvement in VAS, GAD-7 and PHQ-9 scores compared to the Control Group (*p < 0.05*).
04	Li and Pu (2024)	Breast	/	Intravenous; intramuscular;Rectal insufflation	15 ~ 50 μg/mL	Pain; Fatigue	/	Patients in medical ozone treatment showed improvement in fatigue and pain.
05	Tirelli et al. (2023)	Breast	6	Oxygen-ozone autohemotherapy	45 ~ 50 μg/mL	Pain; Fatigue	NRS; Fatigue scoring scale(FSS)	In the experimental group, 66 and 66.26% of patients showed improvement in pain and fatigue, respectively.
06	Clavo et al. (2023b)	Not specified	26	Oxygen-ozone autohemotherapy; Rectal perfusion	10 ~ 30 μg/mL	Pain; Anxiety; Depression	EQ-5D-5L; VAS	18 patients (69%) improved their EQ-5D-5L index after O_3_T with the highest percentage of improvement in “pain or discomfort” (78%) and “anxiety or depression” (79%).
07	Clavo et al. (2023a)	Cervical; Rectal; ovarian; head and neck; lung	16	Oxygen-ozone autohemotherap; Rectal perfusion	10–50 μg/mL	Anxiety; Depression	EQ-5D-5L; HADS	Before MO_3_T: 56% of patients were on anxiolytic and/or antidepressant treatment. After MO_3_T: We found a significant improvement in anxiety and depression.
08	Clavo et al. (2022)	Not specified	18	Rectal perfusion	10–30 μg/mL	Pain	VAS	Before MO_3_T: The median score of VAS was 7 points (range: 5-8points). After MO_3_T: The median score of VAS was 4 points (range: 2-6points).
09	Clavo et al. (2021a)	Pelvic region	6	Rectal perfusion	10 ~ 30 μg/mL	Pain	VAS	The VAS score decreased significantly from 7.8 ± 2.1 at baseline to 2.8 ± 3.8 at 3 months post-therapy (*p* = 0.020).
10	Clavo et al. (2021b)	Pelvic region	6	Rectal perfusion	10–30 μg/mL	Pain	VAS	The baseline pain (7.8 ± 2.1) was significantly reduced at 9 months post-therapy (VAS: 1.7 ± 4.1; *p* < 0.005).
11	Li (2020)	Not specified	100	Oxygen-ozone autohemotherapy	47 μg/mL	Pain	NRS	The combination therapy group (Oxycodone + Ozone) demonstrated a significantly greater improvement in NRS scores.
12	Li and Yao (2018)	Not specified	80	Oxygen-ozone autohemotherapy	47 μg/mL	Pain; Depression	NRS; SDS	Pain was well controlled in both groups with no significant difference in NRS scores, but it showed significantly greater improvement in SDS scores.
13	Zhang et al. (2018)	Not specified	/	Oxygen-ozone autohemotherapy	/	Fatigue	/	Patients in medical ozone treatment showed improvement in fatigue.
14	Tirelli et al. (2018)	/	6	Rectal perfusion	10 ~ 30 μg/mL	Fatigue	VAS	35 patients (70%) achieved a significant improvement (>50% of the symptoms) of fatigue during treatment.
15	Kang et al. (2017)	Liver	100	Oxygen-ozone autohemotherapy	30 μg/mL	Fatigue	/	82% of patients in the experimental group showed improvement in fatigue.
16	Lu et al. (2011)	Breast	60	Oxygen-ozone autohemotherapy	10–15 μg/mL	Fatigue	BFI	The proportion of patients reporting “no fatigue” in the Test Group increased from 0 to 26.67%.

*MO_3_T, Medical Ozone Treatment; NRS, Numeric Rating Scale; FSS, Fatigue Severity Scale; EQ-5D-5L, EuroQol Five-Dimension Five-Level Questionnaire; VAS, Visual Analog Scale; HADS, Hospital Anxiety and Depression Scal; GAD-7, 7-item Generalized Anxiety Disorder Scale. PHQ-9, 9-item Patient Health Questionnaire; SDS, Self-rating Depressive Scale; BFI, Brief Fatigue Inventory.

#### 4.2.1 Tumor site

The application of medical ozone treatment was assessed across a diverse spectrum of malignancies. The evidence base was predominantly derived from studies on breast cancer (*n* = 4 studies), with substantial contributions also from research involving pelvic region malignancies (*n* = 2), and colorectal/gynecological cancers (*n* = 2). Additional evidence was available for head and neck, lung, and lymphoid malignancies, as well as hepatocellular carcinoma. Notably, a significant portion of the included studies (*n* = 6) did not specify the primary tumor site, focusing instead on symptoms like chemotherapy-induced peripheral neuropathy or cancer-related pain that were not site-specific.

#### 4.2.2 Sample of medical ozone treatment in cancer

Our review identified that the current evidence on medical ozone treatment for cancer symptoms is primarily derived from studies with small to moderate sample sizes. As detailed in Table 3, the included studies enrolled a median of 30 participants (range: 6 to 100). While a few trials, such as those by Su et al. (2024; *n* = 86) and Li (2020; *n* = 100), reached a larger scale, the majority (10/16 studies) included 30 or fewer participants. This limitation, compounded by the predominance of case-series designs (9/16 studies) and the significant contribution of evidence from a single research group, fundamentally constrains the generalizability of the findings. Consequently, these factors collectively underscore the critical necessity for future multi-center, large-scale randomized controlled trials to robustly verify the therapeutic efficacy of medical ozone treatment.

#### 4.2.3 Usage of medical ozone treatment

Ozone can be administered through various routes, such as major auto-hemotherapy (MAH), intramuscular, rectal insufflation, or topical application. Among the included references, Oxygen-ozone autohemotherapy was used in most studies, followed by rectal perfusion. There are few studies provide evidence to suggest that any specific method would enhance the therapeutic efficacy of ozone, but it is necessary that an appropriate route of administration should be chosen based on the patient’s condition and medical history.

#### 4.2.4 Dose of medical ozone treatment

According to guidelines from the World Federation of Ozone Therapy (WFOT), medical ozone treatment within the concentration range of 15–50 μg/mL is generally considered safe when administered by trained professionals. The specific dose should be customized based on the patient’s condition and the treated pathology (Li, 2020). The specific dosage within this therapeutic window must be tailored to the individual patient’s condition and the treated pathology. When administered correctly via systemic routes (e.g., autohemotherapy, rectal insufflation), side effects are typically mild and transient, and may include headache, nausea, or local discomfort at the injection site. It is crucial to emphasize that respiratory irritation is exclusively associated with the direct inhalation of ozone gas, a practice that is strictly avoided in standard medical ozone treatment protocols. This route of administration was strictly avoided in all studies included in this review, which utilized systemic methods such as autohemotherapy and rectal insufflation. Consequently, no respiratory adverse effects were reported in the evidence synthesized here.

#### 4.2.5 Symptom management

Analysis of the included literature indicated that medical ozone treatment was primarily used to alleviate a cluster of adverse symptoms in cancer survivors, notably pain, fatigue, anxiety, and depression. Among these symptoms, pain reduction was the most frequently reported improvement following intervention. However, the underlying mechanisms by which medical ozone treatment ameliorates these symptoms remain incompletely elucidated, underscoring the need for further investigation through multi-center, large-scale clinical trials.

## 5 Discussion

The advancement of cancer treatment has prolonged the survival of cancer patients; however, treatment-related adverse effects continue to pose significant challenges (Dawczak-Dębicka et al., 2022). This scoping review, based on 16 included studies, provides a broad overview of the available evidence regarding the use of medical ozone treatment as a complementary therapy for alleviating pain, fatigue, anxiety, and depression in cancer patients. It highlights the scope of existing research and identifies areas where interventions have demonstrated potential benefits. The application of medical ozone introduces a novel approach to adjuvant cancer therapy (Re et al., 2008). It exhibits potent bactericidal properties, enhances red blood cell metabolism, activates the immune and antioxidant enzyme systems, and effectively eliminates inflammatory factors, metabolic waste, and toxic substances. Furthermore, it improves microcirculation, reduces local edema, and enhances tissue oxygen supply (Scassellati et al., 2020). This modality is increasingly recognized in clinical practice. Due to the instability of ozone gas, various administration forms are utilized in oncology, such as ozonated water or oils, major or minor autohemotherapy, and subcutaneous applications (Rowen et al., 2023). The safe dosage of medical ozone generally falls within the range of 15–50 μg/mL, as recommended by international guidelines, and is tailored based on the disease type, location, and patient condition (World Federation of Ozone Therapy (WFOT), 2015). While medical ozone treatment shows promise in cancer care, its potential adverse effects should not be overlooked. Known side effects may include transient symptoms such as headache, nausea, or vomiting; however, respiratory irritation typically occurs only upon inhalation of ozone, which is not a typical route of administration in therapeutic settings. Regulating ozone concentration within a safe and effective range is essential to minimizing risks (İlhan and Doğan, 2021). Currently, literature on the application of medical ozone for symptom management in cancer patients remains limited, with most studies focusing on mechanistic research, safety assessments, and animal experiments. Nevertheless, the potential benefits of medical ozone as an adjunct therapy in oncology warrant attention (Lu et al., 2024). Pain is a major concern for cancer patients and often worsens with disease progression. Medical ozone may help alleviate pain by stimulating the release of enkephalins and scavenging oxygen free radicals in peripheral nerves and the spinal cord (Re, 2024). Cancer-related fatigue is another common and persistent symptom, which can endure for years post-treatment. Medical ozone, rich in oxygen, can improve blood oxygen saturation, inhibit erythrocyte sedimentation, optimize circulation, enhance erythrocyte flexibility, and thereby support physiological function, making it a viable supportive option for managing fatigue (Seledtsov and von Delwig, 2022). Affective disorders such as anxiety and depression are associated with elevated oxidative stress (Martínez-Sánchez et al., 2012). Ozone readily decomposes in the blood to form reactive oxygen species (ROS), which can act as physiological modulators and activate the body’s antioxidant system (Shen et al., 2022). These findings suggest that medical ozone treatment may represent a promising and safe adjunctive strategy in cancer management, though further large-scale clinical trials are necessary to better elucidate its role and anticancer potential.

Currently, guidelines or expert consensus on the application of medical ozone treatment for the management of anxiety, depression, fatigue, and pain in cancer patients are still lacking. It is noteworthy that, although beyond the scope of this review, medical ozone treatment has been mentioned and assigned a recommendation grade in professional guidelines for other specific conditions, such as the management of chronic radiation proctitis (Paquette et al., 2018). This highlights the existing evidence gap between the symptom domains focused on in this review and those with preliminary guideline endorsements, underscoring the need for more high-quality studies to inform future guideline development in our areas of focus.

## 6 Conclusion

In conclusion, medical ozone treatment represents a promising complementary approach for managing specific cancer-related symptoms, particularly pain, fatigue, anxiety, and depression, with a favorable safety profile when administered within established guidelines. Future efforts should focus on integrating it with conventional anticancer therapies to explore novel combined regimens. The current body of international literature remains limited, underscoring a significant research gap that warrants further investigation. Addressing this gap is crucial for meeting the growing need for accessible and low-cost strategies to alleviate these debilitating symptoms in cancer patients.

### 6.1 Study limitations

This scoping review is subject to several limitations, which primarily reflect the current state of the evidence base. First, despite our broad search, the synthesis was deliberately focused on a specific cluster of symptoms (pain, fatigue, anxiety, and depression). Consequently, the application of medical ozone treatment to other cancer-related symptoms falls outside the scope of this review. Second, the body of literature itself is constrained by the small number of available studies, their generally small sample sizes, and the predominance of case-series designs over robust clinical trials. Finally, the fact that a significant portion of the evidence originates from a single research group may affect the generalizability of the findings. These limitations underscore the imperative for future multi-center, large-scale trials to both broaden the symptomatic focus and strengthen the evidence base.

## Footnotes

^1^ https://osf.io/zgpfw/

## References

[ref1] Andrade R. R. Oliveira-Neto O. B. Barbosa L. T. Santos I. O. Sousa-Rodrigues C. F. Barbosa F. T. (2019). Effectiveness of ozone therapy compared to other therapies for low back pain: a systematic review with meta-analysis of randomized clinical trials. Braz. J. Anesthesiol. 69, 493–501. doi: 10.1016/j.bjan.2019.06.007, PMID: 31521383 PMC9391853

[ref2] Bennett M. Paice J. A. Wallace M. (2017). Pain and opioids in Cancer care: benefits, risks, and alternatives. Am. Soc. Clin. Oncol. Educ. Book 37, 705–713. doi: 10.1200/EDBK_180469, PMID: 28561731

[ref3] Bocci V. Borrelli E. Travagli V. Zanardi I. (2009). The ozone paradox: ozone is a strong oxidant as well as a medical drug. Med. Res. Rev. 29, 646–682. doi: 10.1002/med.20150, PMID: 19260079

[ref4] Clavo B. Cánovas-Molina A. Díaz-Garrido J. A. Cañas S. Ramallo-Fariña Y. Laffite H. . (2023a). Effects of ozone therapy on anxiety and depression in patients with refractory symptoms of severe diseases: a pilot study. Front. Psychol. 14:1176204. doi: 10.3389/fpsyg.2023.1176204, PMID: 37599784 PMC10437070

[ref5] Clavo B. Cánovas-Molina A. Federico M. Martínez-Sánchez G. Benítez G. Galván S. . (2025a). Ozone treatment in the Management of Chemotherapy-Induced Peripheral Neuropathy: a review of rationale and research directions. Cancers (Basel) 17:2278. doi: 10.3390/cancers17142278, PMID: 40723162 PMC12293450

[ref6] Clavo B. Cánovas-Molina A. Ramallo-Fariña Y. Federico M. Rodríguez-Abreu D. Galván S. . (2023b). Effects of ozone treatment on health-related quality of life and toxicity induced by radiotherapy and chemotherapy in symptomatic Cancer survivors. Int. J. Environ. Res. Public Health 20:1479. doi: 10.3390/ijerph20021479, PMID: 36674232 PMC9859304

[ref7] Clavo B. Navarro M. Federico M. Borrelli E. Jorge I. J. Ribeiro I. . (2021a). Ozone therapy in refractory pelvic pain syndromes secondary to Cancer treatment: a new approach warranting exploration. J. Palliat. Med. 24, 97–102. doi: 10.1089/jpm.2019.0597, PMID: 32379556

[ref8] Clavo B. Navarro M. Federico M. Borrelli E. Jorge I. J. Ribeiro I. . (2021b). Long-term results with adjuvant ozone therapy in the Management of Chronic Pelvic Pain Secondary to Cancer treatment. Pain Med. 22, 2138–2141. doi: 10.1093/pm/pnaa459, PMID: 33738491 PMC8557383

[ref9] Clavo B. Rodríguez-Abreu D. Galván S. Federico M. Martínez-Sánchez G. Ramallo-Fariña Y. . (2022). Long-term improvement by ozone treatment in chronic pain secondary to chemotherapy-induced peripheral neuropathy: a preliminary report. Front. Physiol. 13:935269. doi: 10.3389/fphys.2022.935269, PMID: 36111149 PMC9468657

[ref10] Clavo B. Rodríguez-Abreu D. Galván-Ruiz S. Federico M. Cánovas-Molina A. Ramallo-Fariña Y. . (2025b). Long-term effects of ozone treatment in patients with persistent numbness and tingling secondary to chemotherapy-induced peripheral neuropathy. A retrospective study. Integr. Cancer Ther. 24:15347354241307038. doi: 10.1177/15347354241307038, PMID: 39797612 PMC11724412

[ref11] Dawczak-Dębicka A. Kufel-Grabowska J. Bartoszkiewicz M. Perdyan A. Jassem J. (2022). Complementary and alternative therapies in oncology. Int. J. Environ. Res. Public Health 19:5071. doi: 10.3390/ijerph19095071, PMID: 35564468 PMC9104744

[ref12] Elvis A. M. Ekta J. S. (2011). Ozone therapy: a clinical review. J Nat Sci Biol Med. 2, 66–70. doi: 10.4103/0976-9668.82319, PMID: 22470237 PMC3312702

[ref13] Götze H. Friedrich M. Taubenheim S. Dietz A. Lordick F. Mehnert A. (2020). Depression and anxiety in long-term survivors 5 and 10 years after cancer diagnosis. Support Care Cancer 28, 211–220. doi: 10.1007/s00520-019-04805-1, PMID: 31001695

[ref14] Guo J. Guo J. Cheng B. Gong M. Sun X. Zhang H. . (2024). Ozone enhances the efficacy of radiation therapy in esophageal cancer. J. Radiat. Res. 65, 467–473. doi: 10.1093/jrr/rrae041, PMID: 38842109 PMC11262864

[ref15] Hayashi K. Onda T. Honda H. Ozawa N. Ohata H. Takano N. . (2019). Effects of ozone nano-bubble water on mucositis induced by cancer chemotherapy. Biochem. Biophys. Rep. 20:100697. doi: 10.1016/j.bbrep.2019.100697, PMID: 31692631 PMC6806368

[ref16] İlhan B. Doğan H. (2021). The authors reply: ozone therapy's efficacy and complications. Am. J. Emerg. Med. 41:255. doi: 10.1016/j.ajem.2020.05.109, PMID: 32563614

[ref17] Kang H. Y. Yang J. Dong J. L. (2017). The effect of medical ozone autotransfusion on basic fibroblast growth factor and alpha-fetoprotein in elderly patients with hepatocellular carcinoma after radiofrequency ablationlderly liver cells after radiofrequency ablation. Mod. J. Integr. Tradit. Chin. West. Med. 26, 3351–3352+3393. doi: 10.3969/j.issn.1008-8849.2017.30.014

[ref18] Koo M. M. Swann R. McPhail S. Abel G. A. Elliss-Brookes L. Rubin G. P. . (2020). Presenting symptoms of cancer and stage at diagnosis: evidence from a cross-sectional, population-based study. Lancet Oncol. 21, 73–79. doi: 10.1016/S1470-2045(19)30595-9, PMID: 31704137 PMC6941215

[ref19] Li X. X. (2020). Clinical observation of medical ozone combined with oxycodone sustained-release tablets in the treatment of moderate to severe cancer pain. Cardiovasc Dis J Integr Tradit Chin West Med (Electron) 8, 40–56. doi: 10.16282/j.cnki.cn11-9336/r.2020.35.027

[ref20] Li Y. Pu R. (2024). Ozone therapy for breast Cancer: an integrative literature review. Integr. Cancer Ther. 23:15347354241226667. doi: 10.1177/15347354241226667, PMID: 38258533 PMC10807353

[ref21] Li H. X. Yao P. (2018). Medical ozone as an adjuvant therapy to oxycodone for moderate-to-severe cancer pain. China J. Mod. Med. 28, 49–53. doi: 10.3969/j.issn.1005-8982.2018.22.009

[ref22] Lu Y. Q. Dong H. M. Yang Y. C. (2011). Nursing intervention for breast carcinoma patients with cancer——related fatigue accepting immuned ozone treatment. Chinese General Practice Nurs. 9, 97–98. doi: 10.3969/j.issn.1674-4748.2011.02.002

[ref23] Lu J. Zheng R. Shi Z. Gao X. Li Y. Fahad A. . (2024). Intracellular Criegee's mechanism-based synergistic ozone therapy mediated by oleogels for cancer treatment. J. Control. Release 370, 879–890. doi: 10.1016/j.jconrel.2024.05.039, PMID: 38782060

[ref24] Martínez-Sánchez G. Delgado-Roche L. Díaz-Batista A. Pérez-Davison G. Re L. (2012). Effects of ozone therapy on haemostatic and oxidative stress index in coronary artery disease. Eur. J. Pharmacol. 691, 156–162. doi: 10.1016/j.ejphar.2012.07.010, PMID: 22796450

[ref25] Paquette I. M. Vogel J. D. Abbas M. A. Feingold D. L. Steele S. R. Clinical Practice Guidelines Committee of The American Society of Colon and Rectal Surgeons (2018). The American Society of Colon and Rectal Surgeons clinical practice guidelines for the treatment of chronic radiation proctitis. Dis. Colon Rectum 61, 1135–1140. doi: 10.1097/DCR.0000000000001209, PMID: 30192320

[ref26] Peters M. D. J. Godfrey C. M. McInerney P. (2020). “Scoping reviews” in JBI manual for evidence synthesis. ed. Joanna Briggs Institute (Adelaide: JBI).

[ref27] Re L. (2024). The molecular key to understanding the medical ozone action. Int. J. Mol. Sci. 25:6148. doi: 10.3390/ijms25116148, PMID: 38892336 PMC11172801

[ref28] Re L. Mawsouf M. N. Menéndez S. León O. S. Sánchez G. M. Hernández F. (2008). Ozone therapy: clinical and basic evidence of its therapeutic potential. Arch. Med. Res. 39, 17–26. doi: 10.1016/j.arcmed.2007.07.005, PMID: 18067991

[ref29] Rowen R. J. Grabovac S. Su T. B. (2023). Ozone dialysis delivers three or more times the ozone than other forms of ozone blood treatment. Med Gas Res. 13, 67–71. doi: 10.4103/2045-9912.356474, PMID: 36204785 PMC9555023

[ref30] Scassellati C. Galoforo A. C. Bonvicini C. Esposito C. Ricevuti G. (2020). Ozone: a natural bioactive molecule with antioxidant property as potential new strategy in aging and in neurodegenerative disorders. Ageing Res. Rev. 63:101138. doi: 10.1016/j.arr.2020.101138, PMID: 32810649 PMC7428719

[ref31] Seledtsov V. I. von Delwig A. A. (2022). Oxygen therapy in traditional and immunotherapeutic treatment protocols of cancer patients: current reality and future prospects. Expert. Rev. Anticancer. Ther. 22, 575–581. doi: 10.1080/14737140.2022.2070153, PMID: 35468308

[ref32] Sen S. Sen S. (2020). Ozone therapy a new vista in dentistry: integrated review. Med Gas Res. 10, 189–192. doi: 10.4103/2045-9912.304226, PMID: 33380587 PMC8092153

[ref33] Shen W. Liu N. Ji Z. Fang H. Liu F. Zhang W. . (2022). Combining Ozonated Autohemotherapy with pharmacological therapy for comorbid insomnia and myofascial pain syndrome: a prospective randomized controlled study. Pain Res. Manag. 2022, 1–10. doi: 10.1155/2022/3562191, PMID: 37214227 PMC10195166

[ref34] Su J. L. Yang L. Long J. P. (2024). High-voltage pulsed radiofrequency combined with medical ozone water for the treatment of acute herpes zoster neuralgia in tumor patients after chemotherapy. Chin. J. Pain Med. 30, 783–787. doi: 10.3969/j.issn.1006-9852.2024.10.011

[ref35] Tirelli U. Cirrito C. Pavanello M. del Pup L. Lleshi A. Berretta M. (2018). Oxygen-ozone therapy as support and palliative therapy in 50 cancer patients with fatigue - a short report. Eur. Rev. Med. Pharmacol. Sci. 22, 8030–8033. doi: 10.26355/eurrev_201811_16432, PMID: 30536352

[ref36] Tirelli U. Valdenassi L. Franzini M. Pandolfi S. Fisichella R. Chirumbolo S. (2023). Oxygen-ozone autohemotherapy in breast cancer patients suffering from fatigue and musculoskeletal pain upon aromatase inhibitors treatment: a case-series study. Eur. Rev. Med. Pharmacol. Sci. 27, 11643–11652. doi: 10.26355/eurrev_202312_34602, PMID: 38095411

[ref37] Tricco A. C. Lillie E. Zarin W. O’Brien K. K. Colquhoun H. Levac D. . (2018). PRISMA extension for scoping reviews (PRISMA-ScR). Ann. Intern. Med. 169, 467–473. doi: 10.7326/M18-0850 30178033

[ref38] World Federation of Ozone Therapy (WFOT) (2015) WFOT guidelines for the safe practice of ozone therapy. Available online at: https://www.wfot.org/ (accessed October 10, 2025).

[ref39] Zhang L. Dong Z. Y. Xu Y. H. . (2018). Research progress on ozone immunotherapy for cancer-related fatigue. World Latest Med Inform (Electron). 18, 25–27. doi: 10.19613/j.cnki.1671-3141.2018.47.013

